# Volatilomic Analysis of Four Edible Flowers from *Agastache* Genus

**DOI:** 10.3390/molecules24244480

**Published:** 2019-12-06

**Authors:** Basma Najar, Ilaria Marchioni, Barbara Ruffoni, Andrea Copetta, Laura Pistelli, Luisa Pistelli

**Affiliations:** 1 Dipartimento di Farmacia, Università di Pisa, Via Bonanno 6, 56126 Pisa, Italy; basmanajar@hotmail.fr (B.N.); luisa.pistelli@unipi.it (L.P.); 2 Dipartimento di Scienze Agrarie, Alimentari e Agro-alimentari, Università di Pisa, Via del Borghetto 80, 56124 Pisa, Italy; 3 CREA Centro di ricerca Orticoltura e Florovivaismo, Corso Inglesi 508, 18038 Sanremo, IM, Italy; barbara.ruffoni@crea.gov.it (B.R.); andrea.copetta@crea.gov.it (A.C.); 4 Centro Interdipartimentale di Ricerca “Nutraceutica e Alimentazione per la Salute” (NUTRAFOOD), Università di Pisa, Via del Borghetto 80, 5614 Pisa, Italy

**Keywords:** VOCs, *A*. ‘Arcado Pink’, *A. aurantica*, *A*. ‘Blue Boa’, *A. mexicana*, essential oil, GC-MS, edible flowers, antioxidant activity, secondary metabolites

## Abstract

Volatilomes emitted from edible flowers of two species of *Agastache* (*A. aurantiaca* (A.Gray) Lint & Epling, and *A. mexicana* (Kunth) Lint & Epling) and from two hybrids (*Agastache* ‘Arcado Pink’ and *Agastache* ‘Blue Boa’) were investigated using a solid-phase microextraction technique as well as the extraction of its essential oils. Oxygenated monoterpenes were almost always the predominant class (>85%) of volatile organic compounds (VOCs) in each sample of *A. aurantiaca*, *A*. ‘Blue Boa’ and *A. mexicana*, with the exception of *A*. ‘Arcado Pink’ (38.6%). Pulegone was the main compound in *A. aurantiaca* (76.7%) and *A*. ‘Blue Boa’ (82.4%), while geranyl acetate (37.5%) followed by geraniol (16%) and geranial (17%) were the principal ones in *A. mexicana*. The essential oil composition showed the same behavior as the VOCs both for the main class as well as the major constituent (pulegone) with the same exception for *A. mexicana*. Total soluble sugars, secondary metabolites (polyphenols, flavonoids and anthocyanins) and antioxidant activity were also investigated to emphasize the nutraceutical properties of these edible flowers.

## 1. Introduction

In the last few decades, the analysis of volatilome has received big attention. In fact, plant volatilome, the aromatic complex of essential oils (EOs) and volatile organic compounds (VOCs), defined as “volatile chemiodiversity” [1], is one of the main traits that enhances our understanding and provides new insights into the physiological processes of plant growth, defense and productivity [2]. Almost all plants are able to emit VOCs, and they are released from leaves, flowers and fruits into the atmosphere, and from roots into the soil [3].

Gas chromatography (GC) in combination with mass spectrometry (GC-MS) is one of the elective techniques for volatilomics [4], the study of the volatilome. Depending on the biological problem as well as the plant material being investigated, analytical procedures for the analysis of volatiles require preparation methods for the extraction and pre-concentration of target compounds [5].

In agreement with the present guidelines aimed on the reduction of the use of organic solvent, both the traditional methods, such hydro-distillation and headspace, were shown to be the main methods used to evaluate volatiles from the plant. Since hydrodistillation was limited to plants producing essential oils, the extraction of VOCs was captured efficiently in the “head space,” an enclosed volume of air surrounding the plant [6], and the use of the Solid Phase Microextraction (SPME) was shown to be the most commonly used method [5], which was a very simple and efficient technique [7] as well as an immediate one-site analysis of biogenic VOC [8]. In fact, this technique, based on the absorption-adsorption of the analytes into a coated fiber, has gained popularity in many fields of analytical chemistry, mainly in food and flavor analysis [9,10,11].

Looking for healthier and functional new foods, people have adopted an interest in edible flowers as creative and innovative ingredients whose popularity and consumption have been increasing worldwide since 1980 [12] due to their powerful and unique tastes, flavors, textures and pigments. The renewed success of edible flowers relies also in their nutritional and healthy properties [13,14]. In fact, several studies have highlighted high quantities of antioxidant molecules [14], primary metabolites [13], minerals [15,16,17,18] and vitamins [13,19,20]. Even though there are no official lists emitted by any international organizations (Food and Agriculture Organization of the United Nations (FAO), World Health Organization (WHO), and European Food Safety Authority (EFSA)), Lim listed over 80 species from about 32 families as edible flowers [21]. Two years later, Lu and his co-workers wrote about 97 families, 100 genera and 180 species, and they specified that the number of edible flowers varies in different countries [14]. In the framework of the INTERREG ALCOTRA Project on edible flowers (INTERREG ALCOTRA ANTEA N°1139, 2014–2020), the *Agastache* genus received special interest due to its good flavor, and so analyzing the chemical composition of its volatiles became relevant.

The *Agastache* genus belongs to Nepetoideae, a subfamily of Lamiaceae (Mint family), and includes 22 species known under the popular name ‘giant hyssop’ [22]. This aromatic genus includes ornamental herbaceous perennial plants, and is almost exclusively native to North America [22,23]. Lot of species is characterized by very pleasant fragrances, similar to anise, mint and licorice [24,25]. Nectar contributes to the sweetness of these flowers and makes them suitable as bee forage [26]. Moreover, some *Agastache* species are medicinal plants, characterized by several biological activities [22].

The evaluation of the flower aroma composition is essential, since it is the second greatest influence on consumer attitudes towards the edible flower’s consumption, only preceded by curiosity [27]. In the last three-decades, the investigation on the *Agastache* volatilome was especially focused on its essential oil (EO) [22]. On the contrary, only two works reported the use of the headspace method for VOC analysis in this genus [28,29].

This work deals with the use of the cited technique for the analysis of four species of the *Agastache* genus: *Agastache* ‘Blue Boa’, *Agastache* ‘Arcado Pink’, *A. aurantiaca* var. ‘Sunset Yellow’ and *A. mexicana* var. ‘Sangria’. These varieties and hybrids were selected due to their ornamental value, aroma and taste (Table 1). As a frame to the volatilome, some classes of antioxidant molecules were quantified, due to their nutraceutical potentials. Soluble sugars were also determined, considering the significant nectar production and the petal’s sweet taste.

## 2. Results and Discussion

The results of the chemical composition of the volatilomes emitted by edible flowers of the four *Agastache* species led to the identification of 67 components (Table 2), representing at least 99% of the total volatiles. Oxygenated monoterpenes were the predominant class in each aroma profile and EO analysis. Their relative percentage ranged between 85.9% in *A. aurantica* and 90.6% in *A*. ‘Blue Boa’ in VOC analysis, and more than 86% in the distilled oils. An exception was noted for the *A*. ‘Arcado Pink’ SPME where the composition of the VOCs was split in three classes; two of them evidenced a similar percentage: sesquiterpene hydrocarbons (38.6%) and oxygenated monoterpene (37.9%) with a good amount of monoterpene hydrocarbons (23.2%).

In detail, the aroma profile of both the *A. aurantica* and *A*. ‘Blue Boa’ flowers showed the same main compound, pulegone, with a percentage of 77.7% and 84.0%, respectively. Pulegone prevailed also in *A*. ‘Arcado Pink’ (36.5%), followed by a good amount of *β*-caryophyllene (20.4%). *A. mexicana* highlighted a different composition, since geranyl acetate was the major constituent (37.5%) followed by both geranial and geraniol with more or less a similar amount (17% and 16%, respectively).

It is worthy to note that the EO composition of this latter species (*A. mexicana*) evidenced the same behavior as already reported for SPME analysis, and confirmed geranyl acetate (61.4%) as the principal compound, whose odor was described as fruity, fatty, sweet, citrus, floral, fresh and lemon-like [30], followed by geranial (11.0%) and geraniol (8.3%).

Notable amounts of pulegone, a compound with a strong pungent aromatic mint smell (IARC Monographs-108), was also detected in the EO composition of the remaining studied species with an amount raging between 79.8% in *A*. ‘Arcado Pink’ to 92.5% in *A. aurantiaca*. These two compounds (pulegone and geranyl acetate) may play a role in the aroma profile and can be perceived by the human olfactory system due to their high amount, even though the main compounds are not always the only ones responsible of the aroma [31].

The volatilome of the *A*. ‘Blue Boa’ inflorescences was already investigated by Wilson and collaborators [28]. Their results were in agreement with what was found herein concerning the class of compounds where oxygenated monoterpenes predominated, with methyl chavicol (estragol) as the major compound (about 40.0% in all the studied samples), while pulegone was the main compound found in this study. Estragol was the unique compound in flower spikes of *A. rugosa* [29] that was reported in the literature.

Flower EOs from two subspecies of *A. mexicana* were investigated by Estrada-Reyes and collaborators [32]. The composition of the *A. mexicana* subsp. mexicana was dominated by estragol (86.78%) and limonene (11.24%), while this latter constituent (limonene 9.49%) together with pulegone (80.07%) characterized the EOs of the *A. mexicana* subsp. xolocotziana. More recently, Kovalenko et al. [33] pointed out methyl eugenol (20.8%), estragol (15.1%) and linalool (12.7%) as main compounds of the same species. However, limonene, methyl eugenol and linalool were present in lesser amounts in the plants studied herein, but pulegone and estragol were completely absent [33]. Going back in the literature revision, the *A. mexicana* aroma composition found here was similar with that reported previously by Svoboda et al. [34], where no estragol was noted in the EOs of this species against an abundance in respect to pulegone (75.3%). Variability in the volatilome among the *Agastache* species was observed and might depend on the harvest time as well as the environmental conditions or cultivation methods [22].

The EO composition of Anice hyssop (*A. foeniculum*) was reported by Ivanov et al. [35], who confirmed the prevalence of estragol (93.45%) together with *β*-caryophyllene (1.2%). This latter constituent represented 2.8% of the total identified fraction in this work. On the other hand, pulegone (43.3%) together with isomenthone (27.2%) prevailed in the same species investigated by Kovalenko et al. [33]. This behavior was similar to the results obtained herein, even though the pulegone amount (82.4%) was 2-fold more abundant, and the isomenthone was of a percentage 13-fold lower (2.1%).

The EOs of three varieties of *A. aurantica* (golde gill) were also reported by Kovalenko et al. [33] who pointed out menthone (53% and 65%, respectively) and pulegone (26% and 25%, respectively) as predominant in the *A. aurantica* varieties ‘Tango’ and ‘Fragrant Delight’, while ‘Apricot Sprite’ evidenced isomenthone (46%) and pulegone (41.4%) as the main ones. The species analyzed in this work were different from all the studied varieties, since pulegone was in higher amount (76.7%), while menthone showed a lesser percentage (5.2%) and isomenthone was lacking.

Pulegone, a monoterpenes hydrocarbon, was produced in a higher percentage by different species of *Agastache* such as *A. rugosa* [36] and *A. scrophulariifolia* (45.2%) [37]. This compound has been reported as Generally Recognized as Safe (GRAS) by the Food and Drug Administration (FDA) since 1965, and it is used as a flavoring agent in food, perfumery and herbal products in the European Union [37,38].

The nutraceutical properties of the edible *Agastache* flowers were investigated, and primary (soluble sugars) and secondary metabolites (polyphenols, flavonoids and anthocyanins) as well as the antioxidant activity were determined (Table 3).

The total polyphenols content (TPC) and total flavonoids (TF) were higher in the two hybrids than in the *A. mexicana* and *A. aurantiaca* varieties. All four flowers showed higher TPC and TF values than other species belonging to *Agastache* genus (*A. foeniculum* and *A. rugosa*) [39], as well as other members of the Lamiaceae family (*Salvia splendens* and *Lamium album*) [20,40,41]. In literature, it was reported that the *Agastache* flowers contain interesting phenolic compounds and flavone glycosides, such as rosmarinic acid, acacetin and tilianin, with variation among different species and cultivars [42]. All these molecules have important biological activities (antioxidant, vasorelaxant, spasmolytic and antinociceptive activities) [43,44,45]. Therefore, more in-depth studies on the plants examined in this work could be interesting to suggest new potential uses of these flowers.

Anthocyanins and carotenoids are the major pigments of petals, producing blue-to-red and yellow-to-red colors, respectively [46]. In this work, the total carotenoid content was relevant in *A. aurantiaca* (198.57 µg/g FW) compared to the other species under evaluation, as highlighted by the yellow colors of its flowers. On the other hand, *A*. ‘Blue Boa’, *A*. ‘Arcado Pink’ and *A. mexicana* are characterized by red-purple petals, so anthocyanins are present in these plants, especially in *A. mexicana*. Moreover, these three flowers are characterized by higher values of anthocyanins than *A. rugosa* [47]. However, the anthocyanins content is relatively low compared to other purple-red colored flowers (e.g., *Monarda dydima*) [20].

Ascorbic acid (vitamin C) is an essential nutrient for humans, as it is not able to be bio-synthesized [48]. The EU Regulation (No 1169/2011) recommended 80 mg as the daily intake for vitamin C (for adults) [49]. The analyzed species showed a very low amount of this compound with the highest value in *A*. ‘Blue Boa’ (3 mg/100 g FW). Therefore, to reach the recommended value, huge amounts of *Agastache* flowers should be consumed. Since few fresh flowers are generally added as ingredient in recipes, the species analyzed in this work cannot help to supply these EU daily requirements. To date, *Tagetes tenuifolia* (241.20 mg/100 g FW) [20] is considered a good source of ascorbic acid, and its flowers contain from 80- to 215-fold more ascorbic acid than the *Agastache* flowers.

Plants belonging to *Agastache* genus can be used in beekeeping [26] to produce a very interesting honey due to their high antimicrobial activity, and are potentially usable in skin products to treat infection caused by bacteria [50]. Nectar is a balanced sugar solution (mainly composed by fructose, glucose and saccharose) that also contains free amino acids, proteins, inorganic ions, lipids, organic acids, phenolic substances and terpenoids, among others [13,51]. In this work, the total content of soluble sugars was in *A. aurantiaca* > *A*. ‘Blue Boa’ > *A. mexicana* > *A*. ‘Arcado Pink’. Moreover, *A. aurantiaca* showed a larger content of glucose, fructose and sucrose compared to the other flowers analyzed in this work. Until now, few studies had reported the total and the reducing sugar content of edible flowers. Grzeszczuk et al. [20] had quantified the percentages of total soluble sugars and sucrose in *Salvia splendens* and *Lavandula angustifolia*, from the Lamiaceae family. Compared with these data, our results highlighted the highest content of these primary metabolites in *Agastache* flowers.

## 3. Material and Methods

### 3.1. Plant Material

All the *Agastache* plants (*Agastache* ‘Blue Boa’, *A. aurantiaca* ‘Sunset Yellow’, *A. mexicana* ‘Sangria’ and *A*.; Arcado Pink’) were provided by the Chambre d’Agriculture des Alpes-Maritimes (CREAM, Nice, France) and were grown at Research Centre for Vegetable and Ornamental Crops (CREA, Sanremo, Imperia, Italy, GPS: 43.816887, 7.758900)—in large pots (30 cm of diameter; 9 L) in a substrate (Hochmoor–Terflor, Capriolo, Brescia, Italy) with a slow release fertilizer (Nitrophoska, Eurochem Agro, Cesano Maderno, Monza e della Brianza, Italy), and were irrigated with a nutrient solution (Ferti 3, Planta-Dȕngemittel, Regenstauf, Germany) every week. The pots were placed in greenhouses with an anti-insect net, and the plants were cultivated using the organic system (without pesticides). In order to avoid damage caused by pests, antagonist insects like *Aphidius colemani* (parasite), *Chrysoperla carnea*, *Adalia bipunctata*, *Phytoseiulus persimilis* and *Amblyseius swirskii* (predators) (Koppert Italia Srl., Bussolengo, Verona, Italy) were released in the greenhouse. *Bacillus thuringensis* subsp. *Kurstaki* (Serbios Srl, Badia Polesine, Rovigo, Italy) was sprayed to control the development of caterpillars. Flowers were picked during their flowering time (summer 2019).

### 3.2. Headspace Trapping for the Floral Set-Up

Inflorescences of each species were accurately weighted (about 0.1 g) and carefully placed separately into 30 mL glass flasks without altering the tissue. The flasks were immediately sealed with aluminum foil and equilibrated at room temperature (around 24 °C) for 1 h.

### 3.3. Sample Analysis: Isolation of VOCs

As recommended by manufacturer’s instructions, a preconditioned 100 μm polydimethylsiloxane PDMS fiber was fitted to a manual sampling fiber holder (Supelco, Bellefonte, PA, USA). The fiber was than exposed to the headspace of the flask containing the samples for 30 min. By the end of the time, the fiber device was transferred into the GC-MS instrument for analysis. The process was repeated twice for each species.

### 3.4. Extraction of Essential Oils

Fresh flowers were hydrodistilled for 2 h using a Clevenger apparatus as recommended by the European Pharmacopeia. The yield of the EOs was very low due to the very low quantity of plant material in disposal (1–2 g). Therefore, the EO was collected with the *n*-hexane grade for HPLC and was immediately injected in the GC-MS.

### 3.5. GC-MS Analysis

Gas chromatography–electron impact mass spectrometry (GC–EIMS) analyses were performed with an Agilent 7890B gas chromatograph (Agilent Technologies Inc., Santa Clara, CA, USA) equipped with an Agilent HP-5MS (Agilent Technologies Inc., Santa Clara, CA, USA) capillary column (30 m × 0.25 mm; coating thickness 0.25 μm) and an Agilent 5977B single quadruple mass detector (Agilent Technologies Inc., Santa Clara, CA, USA). Analytical conditions were as follows: injector and transfer line temperatures 220 and 240 °C, respectively; oven temperature programmed from 60 to 240 °C at 3 °C/min; carrier gas helium at 1 mL/min; injection of 1 μL; split ratio 1:25. The acquisition parameters were as follows: full scan; scan range: 30–300 *m*/*z*; scan time: 1.0 s.

### 3.6. Identification of VOCs and EO Composition

Identification of the constituents was based on a comparison of the retention times with those of the authentic samples, comparing their linear retention indices relative to the series of *n*-hydrocarbons. Computer matching was also used against commercial (NIST 14 and ADAMS 07) [52,53] and laboratory-developed mass spectra libraries built up of pure substances and components of known oils and MS literature data [53,54,55,56,57,58].

### 3.7. Determination of Secondary Metabolites, Ascorbic Acid and Radical Scavenging Activity (DPPH Assay)

Fresh flowers were used to quantify the total carotenoid [59], total polyphenolic content (TPC) (using the Folin—Ciocalteu method, according to Marchioni et al. [60]), total flavonoid (TF) [60] and anthocyanin content (TA) [60]. Radical scavenging activity was determined by the DPPH assay [61], reporting the results as IC_50_ (mg/mL). Total ascorbate (AsA_TOT_) and reduced ascorbate (AsA) were quantified according to the method of Kampfenkel et al. [62], and prior sample extraction is described in Degl’Innocenti et al. [63].

For each analysis, 200 mg of homogeneous samples (three biological replica) were used. The absorbance was read in a UV-1800 spectrophotometer (Shimadzu Corp., Kyoto, Japan).

### 3.8. Sugars Quantification

Fresh flowers (100 mg) were extracted as already described [64], and were thus spectrophotometrically estimated in their soluble sugars content. One mL of the samples was added to 4 mL of 0.2% (*w*/*v*) anthrone solution, and after 30 min of incubation at 90 °C, the absorbance was read at 620 nm. Sucrose, d-fructose and d-glucose determination was performed using a Sucrose/d-Fructose/d-Glucose Assay Kit (Megazyme International Ireland, Co. Wicklow, Ireland) following the manufacturer’s instructions and prior to the extraction described in Tobias et al. [65]. For each analysis, three biological replicas were used.

### 3.9. Statistical Analysis

Data were statistically analyzed by one-way ANOVA followed by Fisher’s probable least-squares difference test with a cut-off significance at *p* ≤ 0.05 (StatView^®^, Version 5.0, SAS^®^ Institute Corporation, Cary, NC, USA).

## Figures and Tables

**Table 1:** Main botanical information and visual appearance of the examined *Agastache* flowers.

Plant Name		Plant Height	Leaves	Flowers	Blossoming Period
*A*. ‘Arcado Pink’	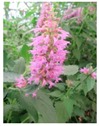	60–70 cm	Medium, opposite, lanceolate, serrated, green/gray color	Purplish/pink in compact ears	May–October
*A. aurantiaca* (*A*. Gray) Lint & Epling, var. ‘Sunset Yellow’	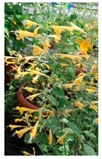	35–40 cm	Small, opposite, lanceolate, serrated, green/gray color	Golden yellow in very loose ears	May–November
*A*.‘Blue Boa’	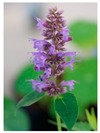	60–70 cm	Medium, opposite, lanceolate, serrated, green/gray color	Dark blue/purple in loose ears	June–October
*A. mexicana* (Kunth) Lint & Epling	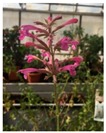	100–120 cm	Large, opposite, lanceolate, serrated, green/gray color	Purple red in very loose ears	June–November

**Table 2:** Volatilome in four species of *Agastache*: *A*. ‘Arcado Pink’ (A.A.P.), *A. aurantica* (A.A.), *A*. ‘Blue Boa’ (A.B.B.) and *A. mexicana* (A.M.).

					SPME				EO			
	Compounds *	Class	LRI	LRI *	A.A.P.	A.A.	A.B.B.	A.M.	A.A.P.	A.A.	A.B.B.	A.M.
					Relative Percentage							
**1**	Furfural	nt	818	836	-	-	-	-	0.2 ± 0.08	-	-	-
**2**	1-Octen-3-ol	nt	979	979	-	-	0.1 ± 0.07	-	-	0.1 ± 0.01	0.2 ± 0.00	-
**3**	*β*-Pinene	mh	981	979	0.1 ± 0.14	-	-	-	-	-	-	-
**4**	3-Octanone	nt	986	984	-	0.1 ± 0.04	0.2 ± 0.00	-	-	-	-	-
**5**	Myrcene	mh	991	991	3.5 ± 0.21	0.4 ± 0.07	0.3 ± 0.21	3.7 ± 0.24	-	0.1 ± 0.01	-	-
**6**	*p*-Cymene	mh	1025	1025	-	-	0.3 ± 007	-	-	-	-	-
**7**	Limonene	mh	1030	1029	17.1 ± 1.70	2.4 ± 0.99	3.6 ± 0.85	0.8 ± 0.10	-	0.5 ± 0.10	0.6 ± 0.14	-
**8**	*cis*-*β*-Ocimene	mh	1038	1037	0.6 ± 0.05	-	-	0.8 ± 0.15	-	-	-	-
**9**	*trans*-*β*-Ocimene	mh	1047	1050	1.0 ± 0.4	-	-	1.3 ± 0.10	-	-	-	-
**10**	*p*-Menta-2,4(8)-diene	mh	1086	1088	0.3 ± 0.14	-	-	-	-	-	-	-
**11**	*p*-Cymenene	mh	1090	1091	-	-	0.1 ± 0.07	-	-	-	-	-
**12**	Linalool	om	1099	1097	-	-	-	1.5 ± 0.33	-	-	-	0.5 ± 0.14
**13**	*trans*-*p*-Mentha-2,8-dienol	om	1116	1113 ^$^	-	-	-	-	2.5 ± 0.06	-	0.3 ± 0.07	-
**14**	1,3,8-*p*-Menthatriene	mh	1119	1110	0.1 ± 0.04	-	0.1 ± 0.40	-	-	-	-	-
**15**	*trans*-*p*-Metha-2,8-dien-1-ol	om	1123	1123	-	-	-	-	3.1 ± 0.06	-	-	-
**16**	*p*-Mentha,1,5,8-triene	mh	1130	1135 ^$^	0.1 ± 0.04	-	-	-	-	-	-	-
**17**	*Neo*-*allo*-ocimene	mh	1131	1132	0.4 ± 0.09	-	-	-	-	-	-	-
**18**	*cis*-*p*-Mentha-2,8-dien-1-ol	om	1138	1138	-	-	-	-	-	-	0.2 ± 0.07	-
**19**	Citronellal	om	1153	1153	-	-	-	-	-	-	-	1.2 ± 0.14
**20**	Menthone	om	1154	1153	-	4.7 ± 0.35	0.4 ± 0.35	-	0.4 ± 0.07	1.4 ± 0.10	0.1 ± 0.00	-
**21**	*iso*-Menthone	om	1164	1163	-	-	-	-	-	0.1 ± 0.01	1.1 ± 0.07	-
**22**	Menthofuran	om	1165	1164	0.5 ± 0.07	1.5 ± 0.64	2.8 ± 1.06	-	-	-	-	-
**23**	Borneol	om	1169	1169	-	-	-	-	-	-	-	0.1 ± 0.00
**24**	*iso*Pulegone	om	1177	1179 ^$^	1.1 ± 0.42	1.9 ± 0.07	2.6 ± 0.92	0.1 ± 0.03	1.1 ± 0.0	2.6 ± 0.20	2.1 ± 0.21	-
**25**	*α*-Terpineol	om	1189	1189	-	-	-	-	0.7 ± 0.08	0.1 ± 0.04	-	-
**26**	Verbenone	om	1204	1205	-	-	-	-	-	0.1 ± 0.10	-	-
**27**	2,6,6-trimethyl,2-Cyclohexen-1-ol	nt	1205	1205 ^$^	-	-	-	-	0.4 ± 0.09	-	-	-
**28**	*n*-Decanal	nt	1206	1202	-	-	-	0.2 ± 0.09	-	-	-	-
**29**	4,7-dimethyl, Benzofuran	pp	1220	1220 ^$^	-	-	0.3 ± 0.05	-	-	-	-	-
**30**	Citronellol	om	1228	1226	-	-	-	6.2 ± 1.48	-	-	-	3.1 ± 0.35
**31**	Pulegone	om	1237	1237	36.5 ± 1,27	77.7 ± 0.42	84.0 ± 5.30	-	79.8 ± 2.15	92.5 ± 0.80	91.8 ± 0.71	-
**32**	*β*-Citral (Neral)	om	1240	1238	-	-	-	3.8 ± 0.30	-	-	-	2.9 ± 0.14
**33**	2-methyl-3-phenyl, Propanal	nt	1244	1244 ^$^	-	-	-	-	-	0.2 ± 0.10	-	-
**34**	Piperitone	om	1252	1253	-	-	-	-	-	0.2 ± 0.11	-	-
**35**	Geraniol	om	1255	1253	-	-	-	16.0 ± 1.86	-	-	-	8.3 ± 0.49
**36**	*α*-Citral (Geranial)	om	1270	1267	-	-	-	17.0 ± 1.56	-	-	-	11.0 ± 0.07
**37**	*iso*Bornyl acetate	om	1286	1286	0.2 ± 0.14	-	-	1.2 ± 0.14	-	-	-	1.1 ± 0.07
**38**	Carvyl acetate	om	1336	1335 ^$^	-	-	0.7 ± 0.57	-	-	-	-	-
**39**	*trans*-Carvyl acetate	om	1337	1342	0.3 ± 0.07	-	-	-	-	-	0.1 ± 0.00	-
**40**	Piperitenone	om	1340	1343	-	0.1 ± 0.07	0.1 ± 0.02	-	1.0 ± 0.04	0.5 ± 0.20	0.6 ± 0.07	-
**41**	*α*-Cubebene	sh	1351	1351	0.1 ± 0.00	-	-	-	-	-	-	-
**42**	Citronellyl acetate	om	1354	1353	-	-	-	2.6 ± 0.56	-	-	-	4.2 ± 0.07
**43**	Neryl acetate	om	1364	1362	-	-	-	1.6 ± 0.17	-	-	-	3.0 ± 0.00
**44**	Geranyl acetate	om	1381	1381	-	-	-	37.5 ± 3.78	-	-	-	61.4 ± 2.40
**45**	*α*-Bourbonene	sh	1384	1384 ^$^	0.3 ± 0.07	-	-	-	-	-	-	-
**46**	*β*-Elemene	sh	1391	1391	0.7 ± 0.35	-	-	-	-	-	-	-
**47**	Methyl eugenol	om	1402	1404	-	-	-	2.0 ± 0.76	-	-	-	1.1 ± 0.00
**48**	*β*-Caryophyllene	sh	1419	1419	20.4 ± 1.27	8.9 ± 1.06	2.5 ± 1.34	2.7 ± 0.96	1.8 ± 0.06	0.6 ± 0.10	0.3 ± 0.00	0.5 ± 0.00
**49**	*β*-copaene	sh	1432	1432	0.6 ± 0.35	-	-	-	-	-	-	-
**50**	*iso*Germacrene D	sh	1448	-	0.2 ± 0.01	-	-	-	-	-	-	-
**51**	*cis*-muurola-4(14),5-diene	sh	1450	1467	0.3 ± 0.00	-	-	-	-	-	-	-
**52**	*α*-Humulene	sh	1454	1455	2.5 ± 0.07	0.9 ± 0.28	0.1 ± 0.07	-	0.3 ± 0.05	0.1 ± 0.08	-	-
**53**	(*E*)-*β*-Farnesene	sh	1457	1457	-	0.5 ± 0.40	-	-	-	0.1 ± 0.05	-	-
**54**	*cis*-Muurola-4(15),5-diene	sh	1463	1459 ^$^	0.1 ± 0.10	-	-	-	-	-	-	-
**55**	Germacrene D	sh	1481	1485	10.9 ± 2,26	0.9 ± 0.57	1.1 ± 0.13	-	4.2 ± 1.04	0.3 ± 0.12	0.4 ± 0.00	-
**56**	Valencene	sh	1492	1496	0.1 ± 0.07	-	-	-	-	-	-	-
**57**	Bicyclogermacrene	sh	1500	1500	0.1 ± 0.02	-	0.4 ± 0.08	-	-	-	0.2 ± 0.07	-
**58**	*α*-Farnesene	sh	1508	1509 ^$^	0.5 ± 0.05	-	0.1 ± 0.05	-	-	-	-	-
**59**	*γ*-Cadinene	sh	1513	1514	0.4 ± 0.07	-	-	-	-	-	-	-
**60**	*δ*-Cadinene	sh	1524	1523	0.6 ± 0.14	-	-	-	-	-	-	-
**61**	*α*-Cadinene	sh	1532	1536	0.1 ± 0.07	-	-	-	-	-	-	-
**62**	Germacrene D-4-ol	os	1574	1576	0.1 ± 0.04	-	-	-	3.9 ± 0.52	0.2 ± 0.12	0.1 ± 0.00	-
**63**	Viridiflorol	os	1591	1593	-	-	-	-	-	0.3 ± 0.00	1.8 ± 0.07	-
**64**	Dodecyl acetate	nt	1609	1610 ^$^	-	-	-	0.2 ± 0.06	-	-	-	-
**65**	*n*-Tetracosane	nt	2400	2400	-	-	-	-	-	-	-	0.5 ± 0.42
**66**	*n*-Pentacosane	nt	2500	2500	-	-	-	-	-	-	-	1.1 ± 0.49
	Unknown				0.2 ± 0.05	0.0 ± 0.00	0.2 ± 0.03	0.8 ± 0.19	0.6 ± 0.07	0.0 ± 0.0	0.1 ± 0.07	0.0 ± 0.00
					**SPME**				**EO**			
	***Class of Compounds***				**A.A.P.**	**A.A.**	**A.B.B.**	**A.M.**	**A.A.P.**	**A.A.**	**A.B.B.**	**A.M.**
	Monoterpene Hydrocarbons (mh)				23.2 ± 1.13	2.8 ± 0.92	4.4 ± 1.34	6.6 ± 0.56	-	0.6 ± 0.10	0.6 ± 0.14	-
	Oxygenated Monoterpenes (om)				38.6 ± 1.98	85.9 ± 1.41	90.6 ± 4.03	89.5 ± 4.96	88.6 ± 3.29	97.5 ± 0.21	96.3 ± 0.21	97.9 ± 0.92
	Sesquiterpene Hydrocarbons (sh)				37.9 ± 2.90	11.2 ± 2.33	4.2 ± 0.97	2.7 ±	6.3 ± 0.89	1.1 ± 0.00	0.9 ± 0.07	0.5 ± 0.00
	Oxygenated Sesquiterpenes (os)				0.1 ± 0.04	-	-	-	3.9 ± 0.52	0.5 ± 0.00	1.9 ± 0.07	-
	Penylpropanoids (pp)				-	-	0.3 ± 0.05	-	-	-	-	-
	Non-terpene Derivatives (nt)				-	0.1 ± 0.04	0.3 ± 0.07	0.4 ± 0.02	0.6 ± 0.08	0.3 ± 0.07	0.2 ± 0.00	1.6 ± 0.09
	**Total Identified**				**99.8 ± 0.21**	**100.0 ± 0.00**	**99.8 ± 0.42**	**99.2 ± 0.35**	**99.4 ± 3.82**	**100.0 ± 0.00**	**99.9 ± 0.02**	**100.0 ± 0.00**

Data are reported as mean values (n = 3; ± SD). * Compounds present with a% > 0.1%; LRI: Linear retention time experimentally determined; LRI*: Linear retention time reported by Adams 2007; ^$^: Linear retention time reported by NIST 2014.

**Table 3:** Determination of secondary metabolites, ascorbic acid, radical scavenger activity and soluble sugars in the four edible flowers belonging to *Agastache* genus. Data are presented as means ± SE (n = 3). Different letters indicate statistically significant differences, at *p* < 0.05 (Fisher’s probable least-squares difference test). Abbreviation: TPC—total polyphenols content; TF—total flavonoids; TA—total anthocyanins; GAE—gallic acid equivalents; CE—±catechin equivalents; ME—malvin equivalents.

Parameters	*A. aurantiaca*	*A*. ‘Arcado Pink’	*A*. ‘Blue Boa’	*A. mexicana*
TPC (mg GAE/g FW)	5.37 ± 0.32 ^b^	7.10 ± 0.25 ^a^	7.34 ± 0.19 ^a^	5.29 ± 0.20 ^b^
TF (mg CE/g FW)	3.04 ± 0.28 ^b^	5.57 ± 0.27 ^a^	4.96 ± 0.19 ^a^	3.52 ± 0.18 ^b^
TA (mg ME/g FW)	0.03 ± 0.00 ^c^	0.17 ± 0.02 ^b^	0.18 ± 0.01 ^b^	0.68 ± 0.02 ^a^
Carotenoids (µg/g FW)	198.57 ± 8.82 ^a^	1.95 ± 0.60 ^b^	11.71 ± 0.40 ^b^	8.69 ± 0.89 ^b^
Reduced ascorbic acid (mg AsA/100 g FW)	1.48 ± 0.06 ^a^	0.79 ± 0.02 ^b^	1.27 ± 0.10 ^a^	1.43 ± 0.08 ^a^
Total ascorbic acid (mg AsA_TOT_/100 g FW)	1.87 ± 0.16 ^b^	1.12 ± 0.02 ^c^	3.06 ± 0.26 ^a^	2.11 ± 0.04 ^a^
DPPH radical scavenging assay (IC_50_ mg/mL)	2.26 ± 0.15 ^a^	1.43 ± 0.04 ^b^	0.86 ± 0.01 ^b^	1.40 ± 0.05 ^c^
Total soluble sugars (mg/g FW)	104.44 ± 2.84 ^a^	56.41 ± 0.67 ^c^	75.81 ± 0.66 ^b^	57.52 ± 0.64 ^c^
d-Glucose (mg/g FW)	16.14 ± 0.67 ^a^	8.13 ± 0.19 ^b^	9.15 ± 0.53 ^b^	6.79 ± 0.32 ^b^
Sucrose (mg/g FW)	17.65 ± 0.44 ^a^	4.53 ± 0.35 ^b^	3.51 ± 0.34 ^b^	2.99 ± 0.20 ^c^
d- Fructose (mg/g FW)	13.25 ± 0.28 ^a^	7.00 ± 0.17 ^b^	6.49 ± 0.25 ^b^	4.92 ± 0.38 ^c^
